# *Rickettsia bellii*, *Rickettsia amblyommii*, and Laguna Negra hantavirus in an Indian reserve in the Brazilian Amazon

**DOI:** 10.1186/1756-3305-7-191

**Published:** 2014-04-17

**Authors:** Lívia de Barros Lopes, Alexandro Guterres, Tatiana Rozental, Renata Carvalho de Oliveira, Maria Angélica Mares-Guia, Jorlan Fernandes, José Ferreira Figueredo, Inês Anschau, Sebastião de Jesus, Ana Beatriz M V Almeida, Valéria Cristina da Silva, Alba Valéria Gomes de Melo Via, Cibele Rodrigues Bonvicino, Paulo Sérgio D’Andrea, Jairo Dias Barreira, Elba Regina Sampaio de Lemos

**Affiliations:** 1 Laboratório de Hantaviroses e Rickettsioses, Instituto Oswaldo Cruz, FIOCRUZ, Rio de Janeiro, RJ 21045-900, Brazil; 2 Fundação Nacional de Saúde, FUNASA-DSEI, Mato Grosso, Brazil; 3 Secretaria Estadual de Saúde, SES, Mato Grosso, Brazil; 4 Laboratório de Biologia e Parasitologia de Mamíferos Silvestres Reservatórios, Instituto Oswaldo Cruz, FIOCRUZ, Rio de Janeiro, Brazil; 5 Programa de Genética, Instituto Nacional de Câncer, Ministério da Saúde, Rio de Janeiro, Brazil; 6 Universidade Federal do Estado do Rio de Janeiro, UNIRIO, Rio de Janeiro, Brazil

**Keywords:** Indian population, Rickettsia bellii, R*ickettsia* amblyommii, Laguna negra virus

## Abstract

**Background:**

The purpose of this study was to identify the presence of rickettsia and hantavirus in wild rodents and arthropods in response to an outbreak of acute unidentified febrile illness among Indians in the Halataikwa Indian Reserve, northwest of the Mato Grosso state, in the Brazilian Amazon. Where previously surveillance data showed serologic evidence of rickettsia and hantavirus human infection.

**Methods:**

The arthropods were collected from the healthy Indian population and by flagging vegetation in grassland or woodland along the peridomestic environment of the Indian reserve. Wild rodents were live-trapped in an area bordering the reserve limits, due the impossibility of capturing wild animals in the Indian reserve. The wild rodents were identified based on external and cranial morphology and karyotype. DNA was extracted from spleen or liver samples of rodents and from invertebrate (tick and louse) pools, and the molecular characterization of the rickettsia was through PCR and DNA sequencing of fragments of two rickettsial genes (gltA and ompA). In relation to hantavirus, rodent serum samples were serologically screened by IgG ELISA using the Araraquara-N antigen and total RNA was extracted from lung samples of IgG-positive rodents. The amplification of the complete S segment was performed.

**Results:**

A total of 153 wild rodents, 121 louse, and 36 tick specimens were collected in 2010. Laguna Negra hantavirus was identified in *Calomys callidus* rodents and *Rickettsia bellii*, *Rickettsia amblyommii* were identified in *Amblyomma cajennense* ticks.

**Conclusions:**

Zoonotic diseases such as HCPS and spotted fever rickettsiosis are a public health threat and should be considered in outbreaks and acute febrile illnesses among Indian populations. The presence of the genome of rickettsias and hantavirus in animals in this Indian reserve reinforces the need to include these infectious agents in outbreak investigations of febrile cases in Indian populations.

## Background

More than 800,000 Indians from 220 ethnic groups speaking 180 languages live in Brazil. Evaluation of indigenous health is very complex, due to demographic and epidemiological differences, especially with some groups that still live in relative isolation without interaction with the prevalent national society [1]. Social changes and economic and environmental factors continue to affect the profile of the Indians’ health, contributing to a higher occurrence of infectious diseases than in other ethnic groups [2,3].

In 2009, the Bureau of Health of the State of Mato Grosso (BHSMT) investigated an outbreak of an unknown acute febrile illness among Indians in the reserve located in the Parecis region, where over 172 cases of hantavirus cardiopulmonary syndrome (HCPS) have been identified since 1999. Blood samples from 59 of 530 apparently healthy Indians were collected; five (8%) were positive for anti-hantavirus IgG antibodies. In addition, as ticks and tick bites were reportedly prevalent in this Indian reserve, serological analysis was performed; IgG antibodies anti-spotted fever group rickettsia (SFGR), *Bartonella* spp., and *Ehrlichia* spp. were detected in sera from 12 (8%), five (8%), and four (6.8%) Indians, respectively. Based on these findings, the objective of this study was to collaborate with the health surveillance system and identify SFGR and hantavirus circulating in arthropods and wild rodents collected in this Indian area.

## Methods

**Figure 1:**
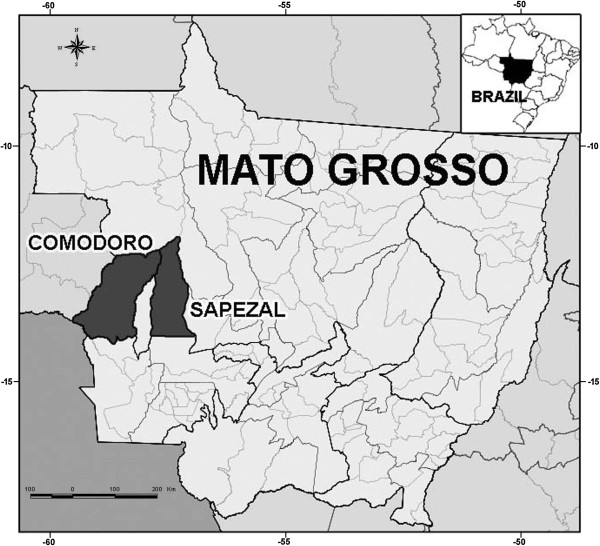
Map of the Mato Grosso state, central-western region of Brazil, showing the municipalities of Comodoro and Sapezal.

In 2010, in response to an outbreak of acute unidentified febrile illness among Indians in the Halataikwa Indian Reserve, northwest of the Mato Grosso state, a study was carried out in two areas of the Parecis micro-region of the Brazilian Amazon in Mato Grosso - municipalities of Comodoro (13° 39’ 46” S 59° 47’ 09” O) and Sapezal (13° 32’ 33” S 58° 48’ 51” O) (Figure 1).

### Arthropod samples

The arthropods were collected from the healthy Indian population and by flagging vegetation in grassland or woodland along the peridomestic environment of the Indian reserve, municipality of Comodoro, by the Indians’ own health service with the collaboration of BHSMT. The identification procedure was done on the basis of morphologic features using standard taxonomic keys [4] and the arthropods were subsequently shipped on dry ice to the Laboratory of Hantaviruses and Rickettsioses, Oswaldo Cruz Institute, FIOCRUZ.

### Small-mammals samples

Eco-epidemiological study was conducted in an area bordering the reserve limits in the municipality of Sapezal, due the impossibility of capturing wild animals in the Indian reserve. The wild rodents were identified based on external and cranial morphology and karyotype [5-7]. Voucher specimens were deposited in the collection of the National Museum, Federal Universidad of Rio de Janeiro, RJ, Brazil. Blood and tissue samples from the wild rodents were obtained in accordance with recommended safety procedures [8], after authorization by the Brazilian Institute for the Environment and Renewable Natural Resources (IBAMA) under license number 60 8054/2008.

### Rickettsia detection

DNA was extracted from spleen or liver samples of rodents and from invertebrate (tick and louse) pools, ranging from one (engorged adult) to 44 (larvae) specimens, by using a QIAamp DNA Mini Kit (QIAGEN, Valencia, CA, USA) following the instructions of the manufacturer. The polymerase chain reaction (PCR) was performed using oligonucleotide primers to amplify the partial citrate synthase gene (*gltA*) of genus *Rickettsia* (RpCS877 and RpCS1258) and outer membrane protein gene (*ompA*) fragments of spotted fever group Rickettsia—SFGR (Rr190-70 and Rr190-602), published previously [9,10].

### Hantavirus detection

In relation to hantavirus, rodent serum samples were serologically screened by IgG ELISA using the Araraquara-N antigen [11]. Total RNA was extracted from lung samples of IgG-positive rodents using Trizol with the Purelink Micro-to-Midi Total RNA Purification System (Invitrogen®, San Diego, CA, USA). In addition, viral RNA was extracted from lung samples of rodents for which there were no blood samples. The amplification of the complete S segment was performed according to Guterres and collaborators [12].

### DNA sequencing and phylogenetic analyses

For DNA purification, the Wizard®SV Gel and PCR Clean-Up System kit (Promega, Corp., Madison, WI, USA) was used according to the manufacturer’s recommendations, and strands were directly sequenced. In the sequencing reaction, the BigDye Terminator™ version 3.1 Cycle Sequencing® Kit (Applied Biosystems) was used according to the manufacturer’s recommendations in an automatic sequencer (Applied Biosystems, ABI PRISM 3130X model, Foster City, CA, USA). Nucleotide sequences were analyzed using MEGA5 software [13], and a consensus sequence was derived from contiguous sequences assembled with the same software.

Multiple sequence alignments were done with sequences obtained from this study and sequences from GenBank using the MUSCLE, in the SeaView version 4 software [14]. The rickettsia phylogenetic tree was constructed using the maximum likelihood (ML) implemented in MEGA5 software. The support for the tree nodes was calculated with 1,000 bootstrap replicates.

Phylogenetic relationships among the hantaviruses were estimated by the Bayesian Markov Chain Monte Carlo (MCMC) method implemented in MrBayes version 3.1.2 [15], using the GTR + G model of sequence evolution, as determined by the jModelTest version 2 [16]. The Bayesian analysis consisted of two simultaneous independent runs of 3 million MCMC generations (burn-in of 25%).

## Results

Two ticks and 121 louse specimens, removed from the asymptomatic Indian population, were taxonomically classified as *Amblyomma* nymphs and *Pediculus humanus* (81 adults and 40 nymphs), respectively. Additionally, 34 ticks were collected by flagging vegetation in grassland or woodland along the peridomestic environment of the Indian reserve—10 *Amblyomma cajennense* (adults), 12 *Amblyomma* larvae, and 12 *Amblyomma* nymphs.

**Figure 2:**
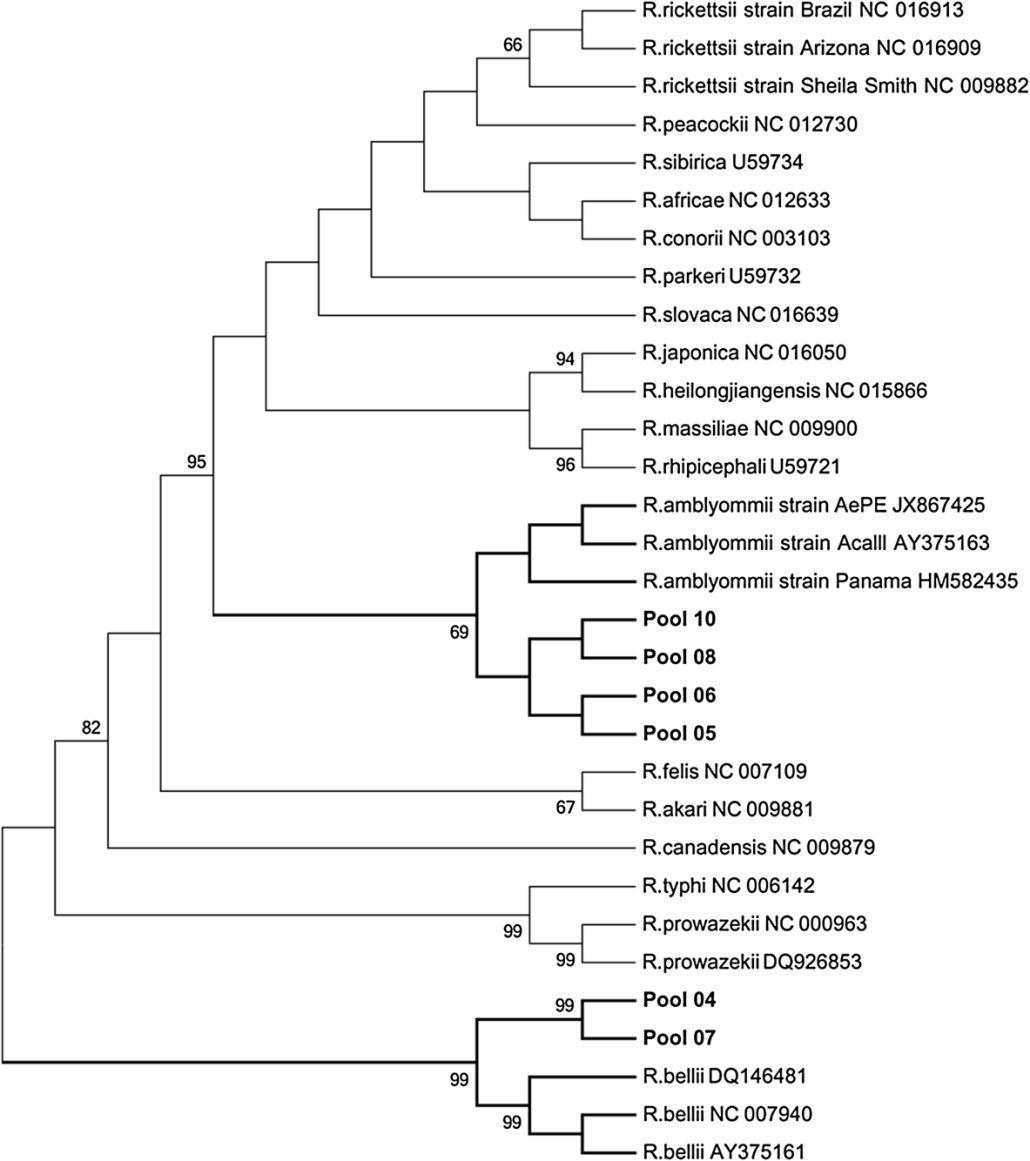
**Maximum-likelihood phylogenetic tree of rickettsia based of the *gltA *gene partial sequence (381nt) and showing the phylogenetic placement of the novel sequences compared with rickettsia associated sequences.** The tree was computed by using MEGA5 (http://www.megasoftware.net). The Tamura 3-parameter model with gamma- distributed rate heterogeneity and a proportion of invariant sites (T92 + G + I) was selected as the best fit evolutionary model according to the Bayesian information criterion calculated with MEGA5. The branch labels include GenBank accession number and species or strain.

**Figure 3:**
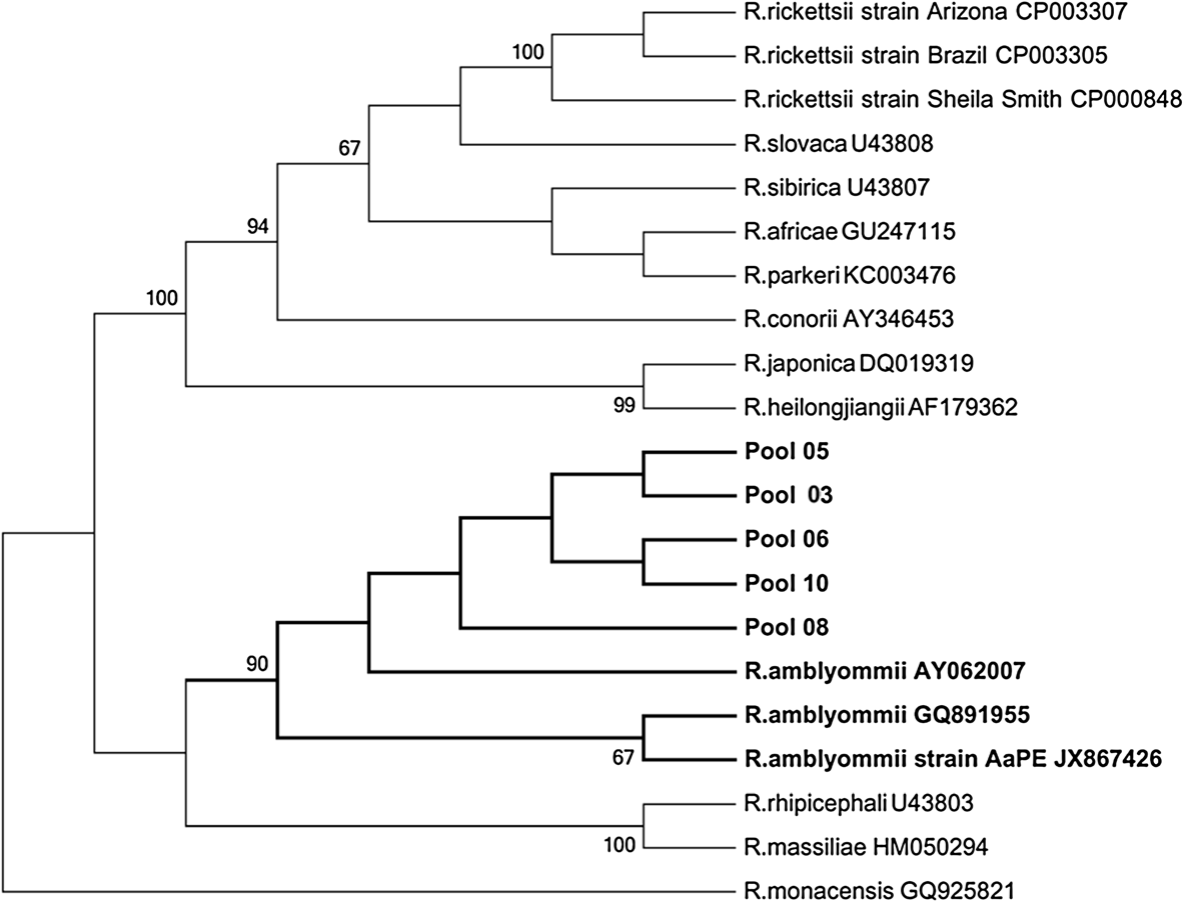
**Maximum-likelihood phylogenetic tree of rickettsia based of the *ompA *gene partial sequence (510nt) and showing the phylogenetic placement of the novel sequences compared with rickettsia associated.** The tree was computed by using MEGA5 (http://www.megasoftware.net). The Tamura 3-parameter model with gamma- distributed rate heterogeneity and a proportion of invariant sites (T92 + G + I) was selected as the best fit evolutionary model according to the Bayesian information criterion calculated with MEGA5. The branch labels include GenBank accession number and species or strain.

Seven of 16 (44%) tick DNA pools were found to be infected with *Rickettsia*; one was composed of *Amblyomma* larvae, two of *Amblyomma* nymphs, and four of *A. cajennense* (adults), all were collected on the ground along the peridomestic environment of the Indian reserve (Table 1). The sequences generated for *gltA* (381bp) and *ompA* genes (510bp) were analyzed using BLASTn searches of GenBank sequences. The DNA sequence analysis of five tick pools had 99% (*ompA*) and 99% (*gltA*) similarity to gene sequences of *Rickettsia amblyommii* [GenBank: GQ891955 and AY375163, respectively] and DNA of two tick pools had 99% (*gltA*) similarity to gene sequences of *Rickettsia bellii* [GenBank: DQ146481]. In the rickettsia phylogenetic tree of the *gltA* gene, the sequences obtained from pools 05, 06, 08, and 10 formed a monophyletic clade with sequences of *R. amblyommii*, while the sequences obtained from pools 04 and 07 formed a monophyletic clade with sequences of *R. bellii* available in the GenBank (Figure 2). In the phylogenetic tree of the *ompA* gene, five tick DNA pools (05, 06, 08, 10, and 03) formed a monophyletic clade with sequences of *R. amblyommii* (Figure 3). All 10 louse DNA pools were PCR negative.

**Table 1:** Molecular analysis of ectoparasites removed from the asymptomatic Indian population and of the environment

**Pools**	**Species (amount)**	**Collected**	***gltA***	***OmpA***
Pool 1	*A. cajennense* (01)	Environment	-	-
Pool 2	*A. cajennense* (01)	Environment	-	-
Pool 3	*A. cajennense* (01)	Environment	-	**Positive**
Pool 4	*Amblyomma* larvae (12)	Environment	**Positive**	-
Pool 5	*Amblyomma* nymphs (06)	Environment	**Positive**	**Positive**
Pool 6	*Amblyomma* nymphs (05)	Environment	**Positive**	**Positive**
Pool 7	*A. cajennense* (01)	Environment	**Positive**	-
Pool 8	*A. cajennense* (01)	Environment	**Positive**	**Positive**
Pool 9	*A. cajennense* (01)	Environment	-	-
Pool 10	*A. cajennense* (01)	Environment	**Positive**	**Positive**
Pool 11	*A. cajennense* (01)	Environment	-	-
Pool 12	*P. humanus* nymphs (08)	Human (Head)	-	-
Pool 13	*P. humanus* nymphs (08)	Human (Head)	-	-
Pool 14	*P. humanus* nymphs (08)	Human (Head)	-	-
Pool 15	*P. humanus* nymphs (08)	Human (Head)	-	-
Pool 16	*P. humanus* nymphs (08)	Human (Head)	-	-
Pool 17	*P. humanus* (04)	Human (Head)	-	-
Pool 18	*P. humanus* (12)	Human (Head)	-	-
Pool 19	*Amblyomma* nymphs (01)	Human (body)	-	-
Pool 20	*Amblyomma* nymphs (01)	Human (body)	-	-
Pool 21	*Pediculus humanus* (10)	Human (Head)		
Pool 22	*P. humanus* (11)	Human (Head)	-	-
Pool 23	*P. humanus* (44)	Human (Head)	-	-
Pool 24	*Amblyomma* nymphs (01)	Environment	-	-
Pool 25	*A. cajennense* (01)	Environment	-	-
Pool 26	*A. cajennense* (01)	Environment	-	-

A total of 153 wild rodents were captured: *Calomys callidus* (65); *Necromys lasiurus* (60); *Oligoryzomys utiaritensis* (9); *Rattus rattus* (8); *Hylaeamys megacephalus* (4), *Oligoryzomys mattogrossae* (1), *Oecomys sp*. (3), *and Calomys sp*. (3). In order to investigate the presence of rickettsial infections in these small mammals, DNA was extracted from spleen or liver samples of 136 rodents for which there were tissue samples. Wild rodents were submitted to molecular analysis for genus *Rickettsia* and were PCR negative to *gltA* and *ompA* genes.

One hundred rodents were submitted to serology for anti-hantavirus antibodies, and one *O. utiaritensis* was reactive against the Araraquara-N antigen. The lung tissue sample of this rodent was submitted to molecular detection (RT-PCR), but it was not possible to recover the viral RNA. In addition, of 53 rodents for which there were no blood samples, one male specimen of the species *C. callidus* was RT-PCR positive. The comparison of the complete S segment with other known hantaviruses showed the highest degree of similarity, at 99% and 98% [GenBank: JX443686 and FJ816031, respectively], with Laguna Negra virus (LANV) from a human case of HCPS from Mato Grosso [17,18]. The sequence obtained was also similar (86%) to LANV [Genbank: AF005727] identified in *Calomys laucha* from Paraguay [19].

**Figure 4:**
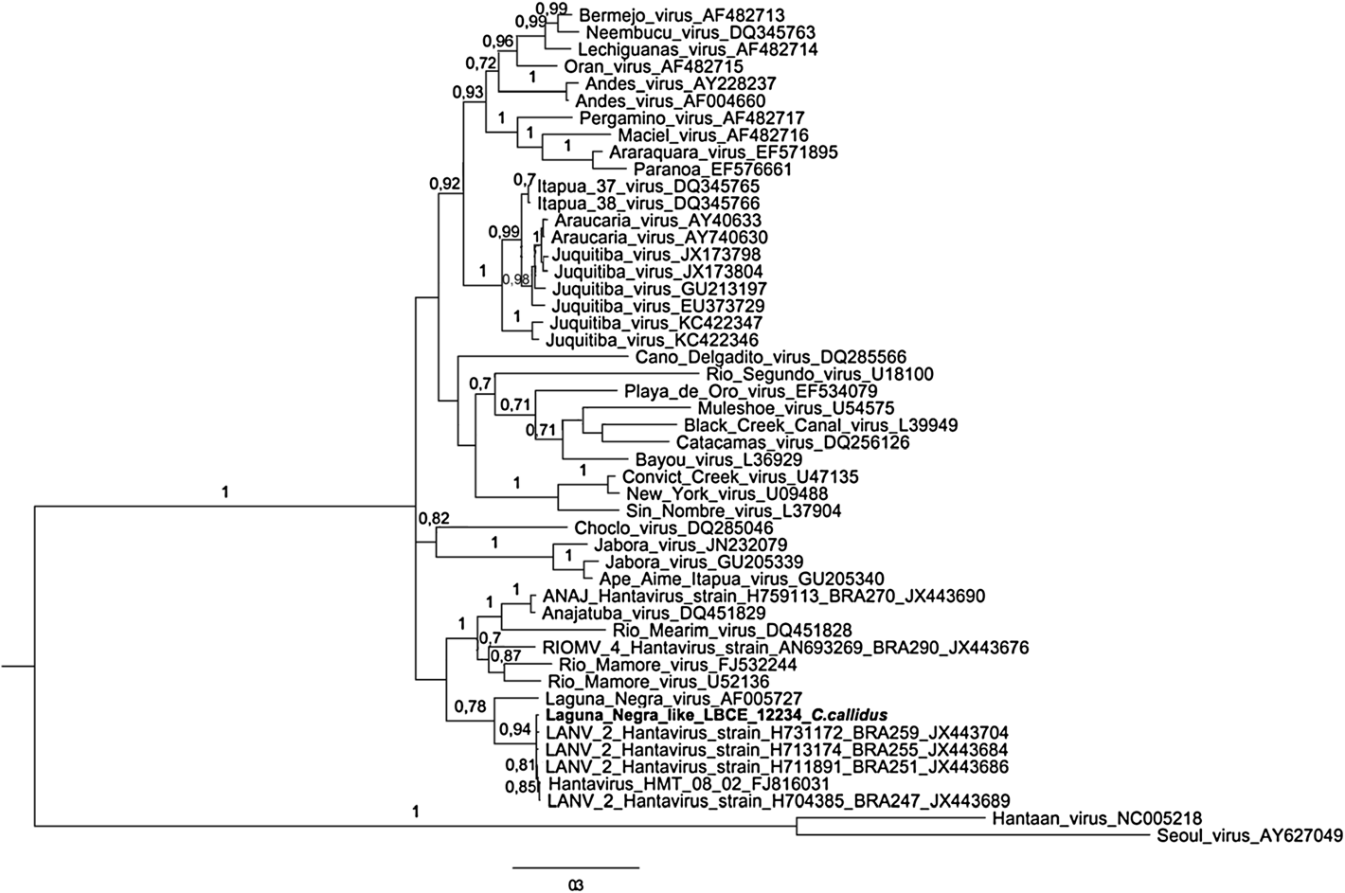
**Phylogenetic relationships among hantaviruses based on Bayesian analysis of genetic distances generated from comparisons of a 950nt fragment of the nucleocapsid gene sequences.** The scale bars indicate an evolutionary distance of 0.3 substitutions per position in the sequence. The numerical value ≥ 0.7 at the node indicates the posterior probability (pp) replicates that supported by the interior branch. The branch labels include GenBank accession number and viral species or strain.

According to phylogenetic inferences, it was possible to check the formation of a well-supported monophyletic clade of our sequence against sequences of LANV available in GenBank. The sequences of LANV from Brazil are closely related to them (Figure 4).

## Discussion

The role of ticks in the transmission of Rocky Mountain spotted fever (RMSF) was first documented by King and also by Ricketts in 1906, and since then several species of ticks have been identified as reservoirs of rickettsiae that are pathogenic and non-pathogenic to humans. In this study, *R. bellii* and *R. amblyommii* genomes were detected in *A. cajennense* ticks collected from the Indian environment. These species of the *Rickettsia* were previously identified in several species of *Amblyomma sp*. ticks in northern Brazil, Peru, and Argentina’s Chaco province [20-23]. Although *R. amblyommii* is considered a non-pathogenic rickettsia, a recent study suggested that this species might be responsible for several cases of RMSF-like disease [24]. As unknown acute febrile illness has been continually reported among Indians in the Brazilian northern region, and as serological tests may have detected cross-reactive antibodies to several SFGR, the possibility of occurrence of human spotted fever in this Indian area should be considered.

*Rickettsia belli*, a member of the ancestral rickettsiae group with unrecognized pathogenicity for humans, have been reported in larvae of *Amblyomma varium* found in infested Amazonian birds caught in Peru [25] and in a host-seeking male of *A. tigrinum* in Argentina, where ixodid ticks collected from vegetation and from humans and wild and domestic mammals in a rural area in the semi-arid Chaco province were found infected with an SFGR of unknown pathogenicity [22]. In this scenario, even if the pathogenicity of *R. bellii* and *R. amblyommii* has not been demonstrated or is unknown, the presence of ectoparasites infected with two species of rickettsiae in the Indian reserve may be a threat.

In relation to HCPS, since its first description in Navajo Indians in the United States, several studies have demonstrated the high incidence of hantavirus infection in the Indian populations throughout the American continent [26]. In 1998, a study conducted in Indian communities in northern Argentina and western Paraguay showed seroreactivity to hantavirus antibodies of 17.1% and 40.4%, respectively [27]. Later, two additional studies on hantavirus infection in the Indian population, also conducted in Paraguay, one in the western region and another in the eastern region, showed prevalence rates of 45.2% and 17.8%, respectively, higher rates than are usually observed in the general population (3.5%, 4.7%). In this sense, their habits, the forests or wild environments in which they dwell, their precarious housing with food storage in places that promote contact with animals and their excreta, among other factors, subject Indians to a greater exposure to hantavirus [28-31].

Our study also revealed the presence of LANV in a *C. callidus* rodent captured in an area bordering the reserve limits in the Sapezal municipality. Genetic analysis revealed the highest degree of similarity, at 99% and 98% with LANV (GenBank: JX443686 and FJ816031, respectively), with human cases of HCPS from Mato Grosso [17,18,32]. The LANV had been first described as a cause of HCPS in the Chaco region, Paraguay, in 1997 in a study that also identified the *Calomys laucha* rodent as the primary reservoir of this virus in Paraguay [19]. In 2004, LANV was identified for the first time in Argentina, recovered from human cases and from *C. callosus* samples. The high sequence identity between human and rodent samples implicated *C. callosus* as the primary rodent reservoir for LANV in Argentina [33]. Subsequently, in 2005, in the city of Concepción, Bolivia, where a non-fatal case of HCPS was reported, LANV was identified in *C. callosus* rodents. The sequencing reaction of the amplified segment showed an 87-88% similarity with LANV and a 99% match with the viral sequences obtained from the patient with HCPS in that region [18]. A recently published study associated LANV with HCPS in Mato Grosso state, Brazil, and cited a previously unidentified potential host, the *C. callidus* rodent corroborating our findings.

In our study, it was possible to perform the sequencing of a complete S segment of LANV in Brazil. The identification of LANV associated with another rodent species of the genus *Calomys* reinforces the idea that many hantaviruses, including LANV, cannot be strictly associated with only one species of rodent reservoir. The capacity of *C. callidus* to harbor LANV and its role as a reservoir are still unclear and new studies need to be conducted in order to better understand the relationship dynamics involving *C. callidus* and LANV.

## Conclusions

Zoonotic diseases such as HCPS and spotted fever rickettsiosis are a public health threat and should be considered in outbreaks and acute febrile illnesses among Indian populations. The interrelationship of the Indian population with rodents and arthropods in forests or wild environments and their precarious housing, among other factors, increases the risk of occurrence of this zoonosis with high mortality rates. Therefore, our study reinforces the importance of the knowledge concerning the geographical distribution and prevalence of zoonotic diseases in Indian reserve areas.

## Competing interests

No competing financial interests exist. The authors have no conflicts of interest or disclosures to make concerning this work.

## Authors’ contributions

JFF, IA, SJ, ABMVA, VCS, AVGMV performed tick and rodents field study and revised the manuscript. LBL and AG lab experiments, processed the data, and drafted the manuscript. TR, RCO, MAMG and JF conducted lab experiments, processed the data, and revised the manuscript. CRB and PSD performed identification of the rodents and revised the manuscript. JDB performed identifications of the arthropods and revised the manuscript. ERSL contributed to study design, field study, data analysis and interpretation, and revised the manuscript. All authors read and approved the final manuscript.
